# Virus Pathotype and Deep Sequencing of the HA Gene of a Low Pathogenicity H7N1 Avian Influenza Virus Causing Mortality in Turkeys

**DOI:** 10.1371/journal.pone.0087076

**Published:** 2014-01-28

**Authors:** Munir Iqbal, Kolli B. Reddy, Sharon M. Brookes, Steve C. Essen, Ian H. Brown, John W. McCauley

**Affiliations:** 1 Avian Viral Diseases Programme, The Pirbright Institute, Compton Laboratory, Compton, Newbury, Berkshire, United Kingdom; 2 Avian Virology, Animal Health and Veterinary Laboratories Agency-Weybridge, Addlestone, Surrey, United Kingdom; 3 Division of Virology, MRC National Institute for Medical Research, Mill Hill, London, United Kingdom; University of Edinburgh, United Kingdom

## Abstract

Low pathogenicity avian influenza (LPAI) viruses of the H7 subtype generally cause mild disease in poultry. However the evolution of a LPAI virus into highly pathogenic avian influenza (HPAI) virus results in the generation of a virus that can cause severe disease and death. The classification of these two pathotypes is based, in part, on disease signs and death in chickens, as assessed in an intravenous pathogenicity test, but the effect of LPAI viruses in turkeys is less well understood. During an investigation of LPAI virus infection of turkeys, groups of three-week-old birds inoculated with A/chicken/Italy/1279/99 (H7N1) showed severe disease signs and died or were euthanised within seven days of infection. Virus was detected in many internal tissues and organs from culled birds. To examine the possible evolution of the infecting virus to a highly pathogenic form in these turkeys, sequence analysis of the haemagglutinin (HA) gene cleavage site was carried out by analysing multiple cDNA amplicons made from swabs and tissue sample extracts employing Sanger and Next Generation Sequencing. In addition, a RT-PCR assay to detect HPAI virus was developed. There was no evidence of the presence of HPAI virus in either the virus used as inoculum or from swabs taken from infected birds. However, a small proportion (<0.5%) of virus carried in individual tracheal or liver samples did contain a molecular signature typical of a HPAI virus at the HA cleavage site. All the signature sequences were identical and were similar to HPAI viruses collected during the Italian epizootic in 1999/2000. We assume that the detection of HPAI virus in tissue samples following infection with A/chicken/Italy/1279/99 reflected amplification of a virus present at very low levels within the mixed inoculum but, strikingly, we observed no new HPAI virus signatures in the amplified DNA analysed by deep-sequencing.

## Introduction

Avian influenza (AI) viruses are divided into subtypes on the bases of the antigenic properties of their two surface glycoproteins, the haemagglutinin (HA) and the neuraminidase (NA). To date, a total of seventeen HA and ten NA subtypes are known, and, with the exception of recently identified H17N10 subtype which was isolated from bats [1], all other AI virus subtypes naturally circulate in wild aquatic birds such as migratory wild waterfowl, gulls and shorebirds [2], [3]. Low pathogenicity (LP) AI viruses from wild birds can become established in domestic poultry and evolve adapting to the new host where infection can result in a range of clinical signs [4]–[6]. Viruses belonging to the H5 and the H7 subtypes are known to be able to evolve to a high pathogenicity (HP) form by acquiring a series of multiple basic amino acids (arginine and lysine residues) at the HA cleavage site [7]. HP forms of AI viruses are differentiated from their LP counterparts by acquiring an ability to replicate in the internal body organs and tissues leading to death due to organ failure [8].Therefore, the differentiation of pathotypes (LP and HP forms) is performed using a combination of intravenous infection of chickens, to assess the clinical disease and define the intravenous pathogenicity index (IVPI) of a virus, and by molecular analysis for presence or absence of a series of basic amino acids at the cleavage site of HA molecule [7], [9]. Whilst LPAI viruses do not cause severe disease in chickens infected experimentally, they are able to cause variable disease signs in other galliforme species [10], [11].

The evolution of LP to HP virus pathotypes of H7 and H5 subtypes has been reported in field and experimental infections in chickens and turkeys [12]–[16]. In some cases, pathogenically distinct subpopulations of viruses may co-exist in the field until a dominant phenotype emerges [17], [18]. In other situations, the same virus may cause varied pathogenicity among different poultry hosts [19]. Viruses of increased virulence have been propagated from samples of LP virus using a number of *in vitro* and *in vivo* procedures; these include continued passage of a LP virus in tissue culture [20]–[22], passage of virus in chick embryos of increased age [23]–[25] and passage of virus in chickens [17], [26], [27]. We have investigated the possibility that a similar selection pressure could have been imposed *in vivo* in turkeys to generate HPAI viruses during infection with a LP chicken-origin virus. We have previously reported on *in vivo* infection of turkeys with a LPAI virus which resulted in severe disease signs and death [28].

To investigate the possibility of virus pathotype evolution over the course of the infection, buccal and cloacal samples collected over the course of infection and tissues harvested from humanely killed birds with clinical signs were analysed for the presence of a molecular signature of HPAI virus. Reverse transcriptase-polymerase chain reaction (RT-PCR) amplicons of the HA gene were analysed by Sanger and Next Generation Sequencing (NGS), and through a sensitive RT-PCR assay.

## Materials and Methods

### Ethics Statement

This study was carried out according to the guidance and regulations of the UK Home Office with appropriate personal and project licences (licence number 70/6201). As part of this process the work has undergone scrutiny and approval by the ethics committee at the AHVLA-Weybridge.

### Virus Stocks

The LPAI H7N1 virus strain A/chicken/Italy/1279/99 (Italy/1279) was isolated during an outbreak of avian influenza in Italy in 1999–2000 (kindly provided by Dr. Ilaria Capua, IZSVe, Padova, Italy). Virus stocks were grown in 9-day-old specified pathogen free (SPF) embryonated fowls’ eggs (Charles River, USA), and virus titres were calculated as median 50% embryo infective dose (EID_50_) according to standard protocols [29], [30]. The IVPI of (Italy/1279) inoculum virus was determined by the standard procedure [31].

### Infection of Turkeys

Infection of turkeys has been described previously [28]. Briefly, infection of turkeys (in groups of 10) housed in Defra approved ACDP-3/SAPO-4, Biosafety Level 3+ (BSL 3+) facilities at AHVLA, Weybridge were inoculated intranasally with doses of 10^2.1^, 10^3.8^, 10^5.2^ or 10^7.4^ EID_50_ of virus delivered in a volume of 0.1 ml. Immediately after inoculation, groups of 10 naïve turkeys were co-housed with those infected with the two lower doses of inoculum. All birds were monitored twice daily for clinical signs, and daily, buccal and cloacal swabs were taken. Any bird that reached a clinical score 4 or 5 (unable to reach food or water), was killed humanely. Some birds died between the intervals of clinical observation. From three selected culled or dead birds tissue samples (brain, heart, spleen, lungs, liver, kidney, intestine and trachea) were collected into 15% w/v brain-heart-infusion broth (BHIB) with antibiotics (penicillin G, 10,000 U/ml; amphotericin B, 20 µl/ml; gentamycin, 1000 µg/ml) for RNA extraction for RT-PCR to examine virus dissemination in internal organs and for in-depth (deep amplicon) sequence analysis.

### Virus RNA Isolation and Determination of Virus Titres in Buccal and Cloacal Swabs

This has been described previously [32]. Briefly, buccal and cloacal swabs in 1 ml of 15% BHIB with antibiotics were re-suspended and briefly mixed before 140 µl of the supernatant was used to extract 50 µl of RNA using Qiagen reagents and a Qiagen robot. The viral titers were determined using influenza matrix gene one-step quantitative RT-PCR. The virus load in buccal and cloacal samples was estimated as relative equivalent units of RNA against 10-fold serial dilution titration of RNA from a known titre of Italy/1279 and presented as the extrapolated titre.

### Virus RNA Isolation from Tissue Samples

Each tissue sample collected in 1 ml BHIB was homogenised as a 10% w/v suspension using the QIAGEN Tissuelyser using 3 mm tungsten-carbide beads (QIAGEN, UK). Virus RNA was extracted from clarified tissue homogenate using the QIA quick RNA extraction kit (Qiagen) according to the suppliers’ instructions.

### Deep Amplicon Sequencing

HA gene sequences were analysed using the deep amplicon sequencing approach described previously [33]. RNA extracted from the inoculum, buccal and cloacal swabs, and tissues were subjected to two-step RT-PCR and cDNA amplicons of HA1 coding region from nucleotide 220–1100 were analysed. Briefly, the RT-reactions were performed using the Verso™ cDNA Kit (Thermo Scientific) according to the suppliers’ instructions with an influenza virus universal oligonucleotide primer (5′-AGCAAAAGCAGG-3′). Each RT reaction was then amplified with PfuUltra™ II fusion HS DNA polymerase (Stratagene) and H7 HA specific primers (primer sequences are available on request), ligated into the pCR-Blunt vector (Invitrogen) and transformed into TOP10 *E. coli* (Invitrogen). Positive bacterial colonies were sequenced commercially (GATC Biotech, Constance, Germany) using the ABI 3730 XL (Applied Biosystems) Sanger sequencing platform.

### Differential Real-time PCR (dRT-PCR)

**Figure 1:**
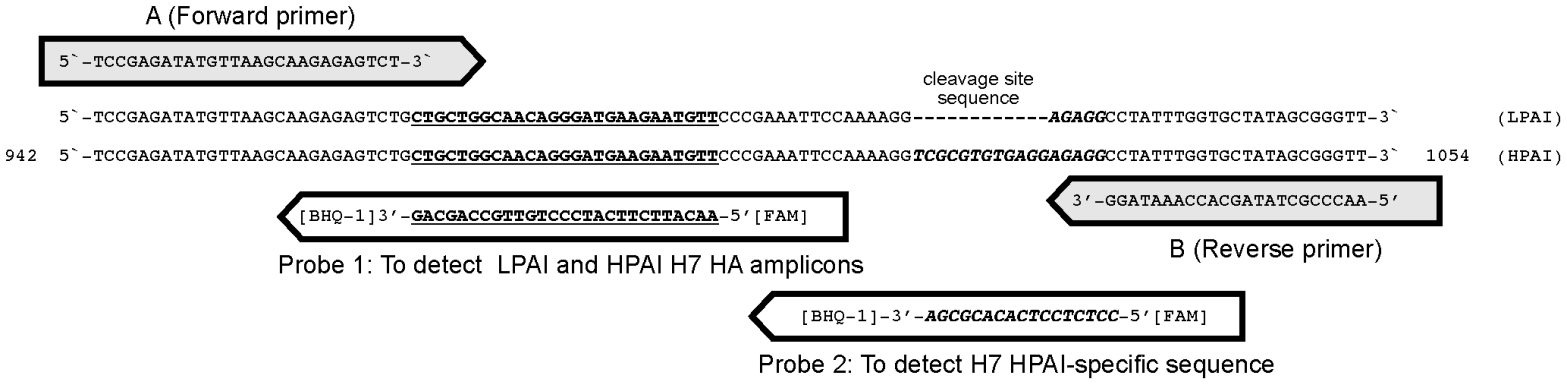
Design of oligonucleotides for dRT-PCR for detection of HPAI virus sequences in H7 subtype viruses. The sequences for (A) the common forward primer, (B) the common reverse primer, (Probe 1) a common probe for quantification of both HPAI and LPAI H7 virus HA amplicons and (Probe 2) a probe to detect the specific HPAI virus amplicon are shown. The sequences corresponding to the virus HA genes are from LPAI and HPAI viruses isolated during the H7N1 avian influenza virus outbreak in turkeys, 1999–2000. Numbering refers to the HPAI virus nucleotide sequence beginning from HA gene start codon.

To detect LPAI and HPAI virus, a differential real-time PCR (dRT-PCR) was employed in which two parallel PCR reactions on a cDNA template prepared from tissue and inoculum samples (described above) were set up using forward (5′-TCCGAGATATGTTAAGCAAGAGAGTCT-3′) and reverse (5′-AACCCGCTATAGCACC AAATAGG-3′) primers with two different probes. One ([FAM]-AACATTCTTCATCCCTGTTGCCAGCAG-[BHQ-1] was designed to detect both LPAI and HPAI virus sequences by hybridising to the HA coding region from nucleotides 970 to 996 of the H7 HA gene. The other was an “HPAI virus-specific” probe ([FAM]-CCTCTCCTCACACGCGA-[BHQ-1]) was designed to detect specifically HPAI virus sequences conserved in the HA of H7N1 HPAI viruses isolated from Italy during 1999–2000, and detects nucleotides 1015 to 1031 of these viruses (Figure 1). For both assays, a master-mix was prepared by diluting 2x TaqMan® Fast universal PCR Master Mix (Applied Biosystems) in water containing 0.5 µM of each forward and reverse primers, and 0.25 µM of either the H7 HA common probe or the HPAI virus-specific probe. PCR cycling (40 cycles; 94°C for 15 s and 60°C for 1 min) was performed on an ABI 7500 Fast Real-time PCR system (Applied Biosystems); all standards, test samples, and controls (positive, negative and extraction control) were analysed in triplicate.

### Ultra-deep Amplicon Sequencing

Ultra-deep sequence analysis of the HA gene cleavage site was performed using RNA samples extracted from Italy/1279 inoculum stocks and tissues (trachea, brain, and liver) from infected turkeys. HA gene amplicons of 235 bp (bases 937–1171 of the HA open reading frame) were prepared by two-step RT-PCR described above. Briefly, the RT-step was performed using the Verso™ cDNA Kit (Thermo Scientific) with influenza virus universal oligonucleotide primer (5′-AGCAAAAGCAGG-3′) and the PCR step was performed using PfuUltra™ II fusion HS DNA polymerase (Agilent technologies) with a forward primer (5′-cgtatcgcctccctcgcgccatcag-NNNNNNNNNN-AAATGTCCGAGATATGTTAAGCAAGA-3′) consisting of a universal adapter sequence (primer A), indicated in lower case, a multiplex identifier sequence (MID tag, consisting of 10 bases of known sequence for each sample, indicated above as N in the sequence of the primer) and Italy/1279 HA specific sequence of 26 nucleotide bases (937–962). The reverse primer (5′-ctatgcgccttgccagcccgctcag-ATTTCCTGTTACTTGATCAATTG-CTG-3′) contained universal reverse primer B sequence (indicated in lower case) followed by Italy/1279 HA specific sequence of 27 bases (1171–1145). PCR amplification was performed in a T100™ thermal cycler (Bio-Rad) programmed for the initial denaturation step (95°C for 4 min), followed by 35 cycles of amplification (95°C for 20 s, 58°C for 30 s, 72°C for 30 s) and a final extension (72°C for 10 min). PCR products were purified using gel electrophoresis and recovered using a gel extraction kit (QIA quick, Qiagen). After quantification with Quant-iT™ Picogreen® dsDNA assay kit (Invitrogen), all PCR products generated from each tissue sample were mixed together in equimolar concentrations and subjected to emulsion PCR using the GS FLX Titanium (Lib-A) MV emPCR kit (Roche Applied Science) according to the manufacturer’s instructions. Unidirectional sequencing of the amplicons from adapter A was carried out on a quandrant of a PicoTitrePlate on the Roche Genome Sequencer FLX (GS FLX) platform. The sequence data of amplicon reads was submitted to the European Nucleotide Archive (ENA).

## Results

### LPAI Virus Inducing High Mortality in Turkeys

The IVPI of hen’s egg-derived Italy/1279 inoculum was determined in six-week-old chickens. None of the inoculated chickens showed any signs of disease throughout the 10 days of observation and the results established that the selected virus inoculum prepared to infect turkeys had an IVPI value of 0.0.

**Figure 2:**
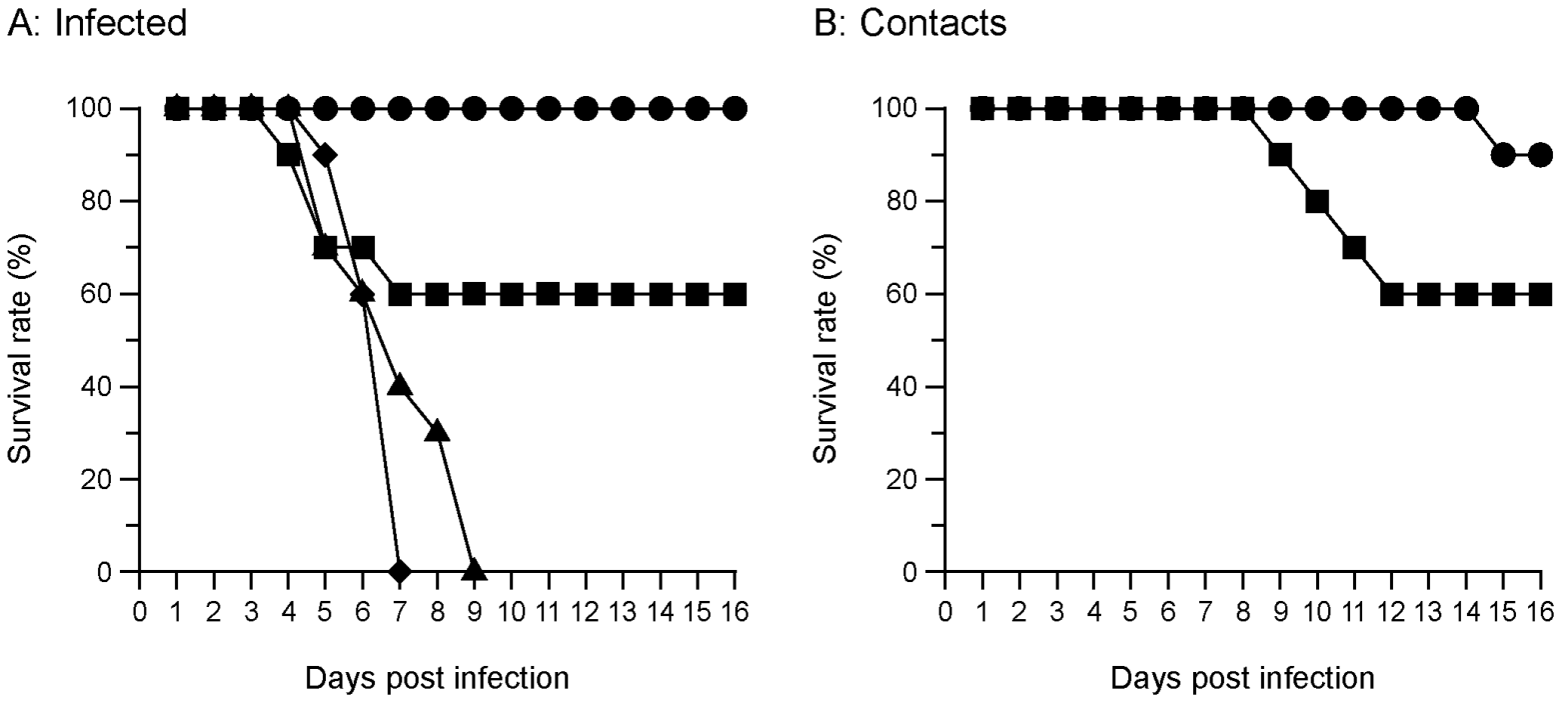
Survival of turkeys infected with Italy/1279 virus. (A) Infected turkeys that were inoculated with Italy/1279 virus doses of (•) 10^2.1^, (▪) 10^3.8^, (▴) 10^5.2^, (♦) 10^7.4^ EID_50_. (B) Contact turkeys were housed with the inoculated turkeys that received inoculum doses of (•) 10^2.1^ EID_50_ and (▪) 10^3.8^ EID_50_ of Italy/1279 stock.

We have reported that groups of turkeys infected at low, medium and high doses, resulted in considerable variation in virus shedding and death (Table S1, showing virus shedding profiles in turkeys inoculated with 10^5.2^ EID_50_, shedding profiles for other doses of inoculum have been described previously [28]). The infected birds showed a variety of clinical signs, ranging from general depression, reluctance to feed, respiratory distress, weight loss, poor coordination and torticollis from day 1 post infection (pi). No death was recorded in the lowest virus dose group (10^2.1^ EID_50_) and all birds had recovered from infection within 16 days pi. In contrast, in groups infected with 10^5.2^ and 10^7.4^ EID_50_ doses of the virus, all birds died or were culled for welfare reasons. In the 10^3.8^ dose group, four (40%) birds died and the remaining six birds survived until the end of the experiment. In the groups that received 10^5.2^ and 10^7.4^ EID_50_ virus dose, all birds died or were culled as a result of influenza infection (Figure 2A). Transmission of virus from directly infected birds to naïve contact birds in the groups infected with the two lowest doses of virus was examined. Of the 20 naïve contact birds (10 per dose group), all were infected and five died or were culled *in extremis* (Figure 2B).

Tissue samples were taken from three birds: a turkey (#83) that was directly infected and found dead on the fourth day after infection with 10^3.8^ EID_50_ of virus, from a contact turkey (#95) that died on the eighth day following the first detection of virus from the same dose group (Table S2, showing virus shedding profiles of these two turkeys, the shedding data of these turkeys have been described previously) [28]., and from a turkey (#22) that was culled for welfare reasons on the ninth day following infection with 10^5.2^ EID_50_ (Table S1).

### Characterisation of HA Cleavage Site by In-depth Sequence Analysis of Virus RNA Recovered from Infected Turkeys

We considered three possible reasons for death in turkeys infected with the LPAI H7N1 virus. First, the birds died of LPAI virus infection, second, there was evolution of a LPAI virus to a HPAI virus within the infected host, or third, there was heterogeneity of virus within the original virus inocula with a small population of HPAI virus in the LPAI virus stock. To address these options samples from designated tissues from the three selected infected birds were assessed by deep amplicon sequence analysis using standard Sanger sequencing of cDNA clones prepared from the amplicons. The sequences of these cDNA amplicons were compared to the sequences of the virus RNA in the inoculum and in buccal and cloacal swabs, as previously reported [28] by supplementing an additional sequences of cDNA clones made from the inoculum, buccal and cloacal swab samples to increase the sensitivity of the analysis. From the inoculum stock 180 clones, and from buccal and cloacal swab samples a total of 3592 clones were produced and analysed (Table 1). Of this total, 1107 clones were analysed from swabs collected each day during the course of infection from two birds (turkey #83 and #95) that died following infection; 116 clones were made from tissue samples (trachea, 34; lungs, 25; intestine, 9; liver,10; kidney, 14; spleen, 24) collected after death (Table 2). The deduced amino acid sequence of a total of 3708 cDNA clones prepared from these samples taken from the infected turkeys showed no evidence of the presence of a HPAI virus signature sequence. Using the estimates of Cannon and Roe [34] to assess the sensitivity of the detection of a minor species it was estimated that there was a 95% probability of detecting a HPAI virus in the swab samples should HPAI virus be present at a prevalence of 0.12%. For tissue samples, HPAI virus would be detected with 95% probability should it be present at a level of 2.5% of the total virus population. We concluded that the prevalence of HPAI virus from *in vivo* tissues from severely affected birds was below 2.5% and perhaps considerably lower.

**Table 1:** Number of clones sequenced from inoculum and from swabs collected at each time point from turkeys infected with Italy/1279 H7N1 virus.

Swabs	Number of clones sequenced at each time point (number of birds sampled)									Total number of clones analysed	Clones with cleavage site identical to LPAI virus
Days pi*	1	2	3	4	5	6	7	8	9		
Inoculum										180	all
Buccal	190 (9)	189 (2)	1191 (18)	134 (8)	86 (1)	159 (6)	182 (1)	88 (1)	769 (15)	2988	all
Cloacal	–	–	319 (4)	43 (3)	–	16 (1)	–	–	226 (7)	604	all

Clones containing HA gene cDNA amplicons made from selected swab samples (described in tables S1, S2 and [28]) were analysed using Sanger sequencing ABI 3730 XL DNA analyser.

* Days post infection.

**Table 2:** Deep amplicon sequence analysis of tissue samples from birds that died from infection with Italy/1279 H7N1 virus.

Bird ID	Tissue samples analysed						Total number of clones analysed	Clones with cleavage site identical to LPAI virus
	Trachea	Lungs	Kidney	Spleen	Liver	Intestine		
83	16	14	11	16	10	–	67	all
95	18	11	3	8	–	9	49	all

Clones containing HA gene cDNA amplicons made from tissue samples were analysed as described in Table 1.

### Differentiation of LPAI and HPAI Viruses by Real-time RT-PCR

To explore further the possibility that HPAI virus might have been present at a lower level within the samples, we designed a dRT-PCR protocol for detection of HPAI virus in samples from LPAI virus infected birds. To set up the assay to detect HPAI virus contaminating sequences in the LPAI virus inoculum the assumption was made that either the virus could be contaminated with HPAI virus from the Italian outbreak or that if evolution occurred in the turkey host it could represent the sequences that emerged during the Italian outbreak of 1999–2000. The HPAI virus sequence-specific dRT-PCR was, therefore, based on the KGSRVRR*GLF sequence in HPAI virus isolates from the Italian outbreak (of 31 HA gene sequences from HPAI viruses collected during the epizootic available in public databases, all encoded this sequence with a small degree of non-synonymous polymorphism at a single residue, representing 6.5% variation). The LPAI and HPAI virus dRT-PCR assays was designed to be able to detect both LPAI and HPAI H7 HA sequences representative of viruses from the Italian outbreak using two Taqman probes analysed in parallel reactions. The amplicon for analysis spanned the HA1/HA2 cleavage site; one probe was designed to detect a conserved region in both LPAI and HPAI viruses in close proximity to the HA1/HA2 cleavage site; while the second probe was designed to detect, in the same amplicon, the additional 12 nucleotide insertion observed during evolution from LPAI to HPAI viruses in Italy in 1999–2000 outbreak (Figure 1).

**Figure 3:**
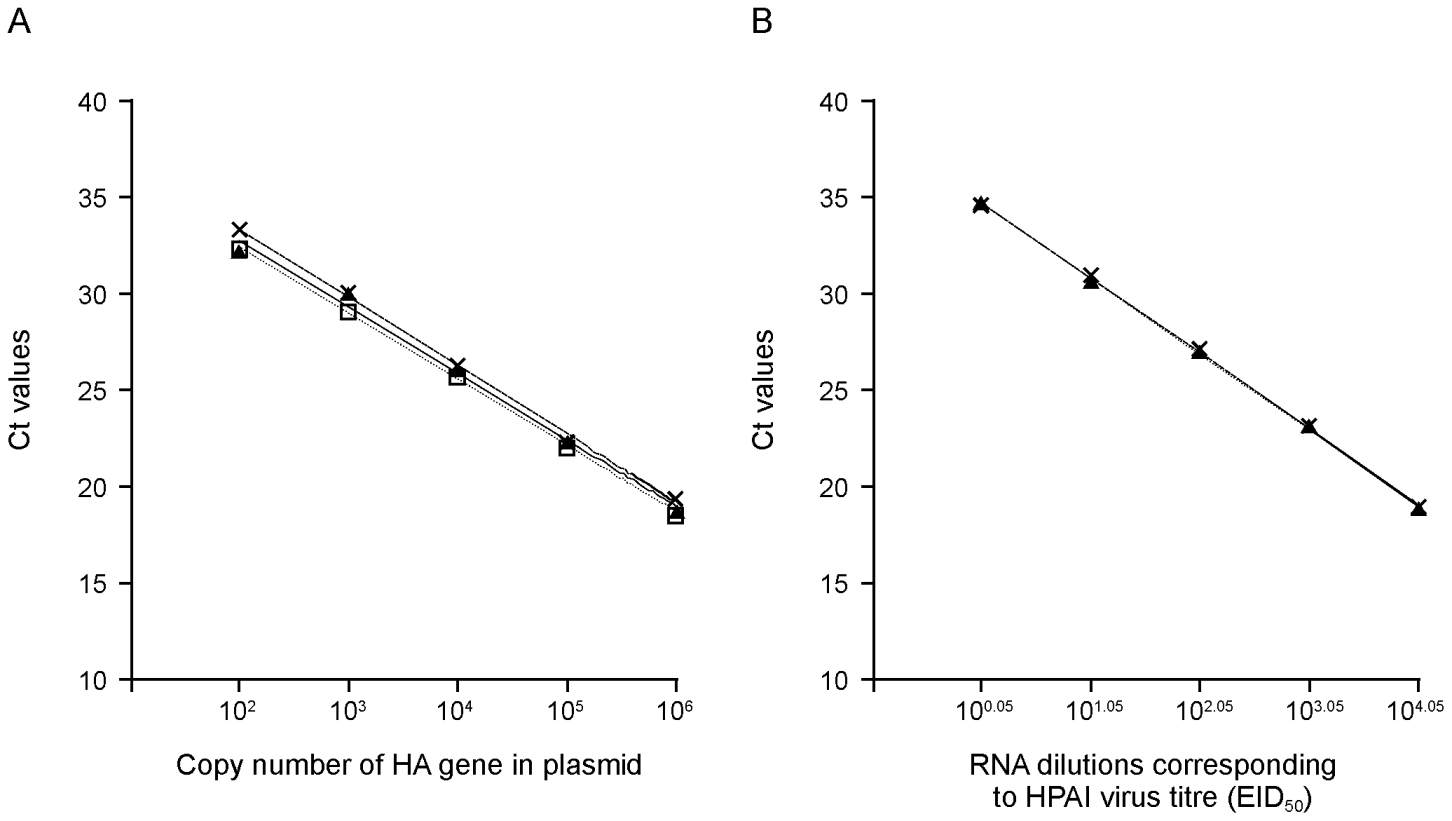
Estimation of sensitivity and amplification efficiencies of dRT-PCR assays to detect HPAI virus in mixed populations of nucleic acid containing LPAI and HPAI virus RNAs. (A) Standard curves generated from reactions performed in triplicate PCR reactions with a dilution series of plasmid constructs: (×) pZb984-HA-HPAI virus DNA (containing the HPAI virus HA cDNA corresponding to A/ostrich/Italy/984/00 that carries an additional four amino acids (SRVR) at the HA1/HA2 cleavage site) detected by HPAI virus- specific probe, (□) pZb984-HA-HPAI virus DNA detected by H7 HA-common probe, or (▴) pZb1279-HA- LPAI virus DNA (a construct containing the HA gene cDNA corresponding to Italy/1279) detected by the H7 HA-common probe. (B) The effect of addition of cDNA reverse transcribed from LPAI virus RNA to the cDNA prepared from HPAI virus RNA. (×) A constant amount of LPAI (Italy/1279) virus RNA corresponding to 10^4.85^ EID_50_ virus titre or (▴) water was added to a 10-fold dilution series of RNA extracted from the HPAI virus (A/ostrich/Italy/984/00) infectious allantoic fluid containing 10^6^ EID_50_ virus/0.1 ml. The lines were obtained by linear regression analysis and represent the mean Ct values from three assays.

The sensitivity of this assay was investigated by testing a series of dilutions of plasmids containing nucleotides 9–1130 from the A/ostrich/Italy/984/2000 (HPAI) virus and the equivalent region from the Italy/1279 (LPAI) virus. The amplification of 10-fold dilutions of HPAI and LPAI virus DNA detected by either the HPAI-specific probe or the H7 HA-common probe reproducibly displayed a linear relationship between Ct (cross threshold) values and HA copy number with a correlation coefficient (R^2^ value) of 0.99. This reaction efficiency was calculated to detect reliably as few as 100 copies of the HA gene per reaction mixture (Figure 3A). Whether the presence of RNA from a LPAI virus might affect the efficiency of the amplification or detection of the HPAI–specific sequences with the HPAI virus probe was tested. The results presented in figure 3B clearly demonstrated that the presence of LPAI cDNA (equivalent to a virus titre of 10^4.85^ EID_50_) in HPAI-specific detection reaction samples did not interfere with the HPAI virus cDNA detection specificity or sensitivity, with the slopes and intercepts of the graphs being identical. The quantification showed that the system had a detection limit similar to the RNA levels present in 10^0.05^ EID_50_ virus titres (Figure 3B). From these data we concluded that the HPAI virus RNA-specific dRT-PCR was able to detect 100 copies of HA gene per reaction mixture and the presence of LPAI virus RNA did not show any interference with the HPAI virus detection specificity. The results of dRT-PCR analysis of the inoculum indicated that this stock did not contain detectable levels of contaminating HPAI virus.

**Figure 4:**
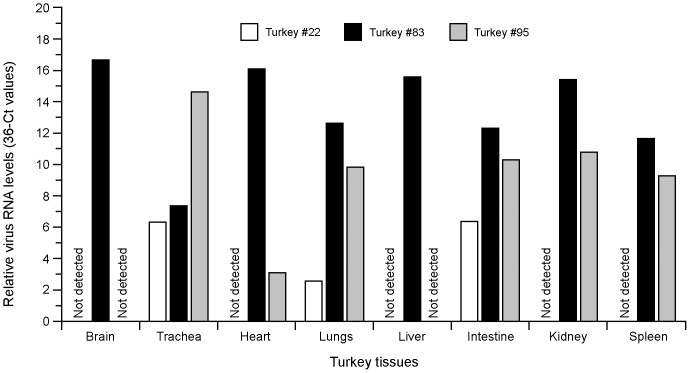
Detection of the HA gene of Italy/1279 (H7N1) LPAI virus in tissues taken from three turkeys which died or were culled after succumbing to infection. The levels of virus RNA were analysed in triplicate by the differential H7N1 HA gene specific-PCR as described in the materials and methods using a standard curve for the HA gene (9–1130 bp) containing plasmids pZb1279-HA-LPAI virus or pZb984-HA-HPAI virus. White bars - tissue samples taken from the turkey #22 that had severe disease and culled for welfare reasons on the ninth day pi in an independent experiment, black bars - tissue samples taken from a turkey #83 found dead on the fourth day pi., grey bars - samples taken from a contact bird #95 that died on the eighth day following the first detection of virus in this animal, not detected - levels of virus RNA were below dRT-PCR detection limit.

The levels of virus RNA in the tissue samples indicated that all three birds analysed showed the presence of virus RNA in the trachea, lungs and intestine (Figure 4). One of the three birds also had detectable virus RNA in the kidney, heart and spleen (#95) and the third bird examined, which died by the fourth day after infection, had virus in all the organs examined: in the brain, trachea, heart, lungs, liver, intestine, kidney and spleen (#83) (Figure 4). Although the common HPAI and LPAI virus probe was able to detect viral RNA in tissues, in no case any signal for the exclusive HPAI virus probe was detected.

### HA Cleavage Site Analysis of Virus RNA Recovered from Turkeys by Ultra-deep Sequencing

An additional analysis was undertaken to address the possibility that a minor population of HPAI viruses still may exist that were not detected by dRT-PCR because the assay, as designed, would only detect HPAI virus nucleotide sequences at the HA1/HA2 cleavage site that were identical to HPAI H7N1 viruses isolated from Italy in 1999/2000. The HA cleavage site sequence in virus RNA samples recovered from tissue samples (trachea, liver and brain) from three turkeys and from inoculum stock (Table 3) were characterised in a sequence-independent manner. cDNA amplicons of 235 bp extending from nucleotides 937 to 1171 and containing coding region of HA1/HA2 cleavage site were prepared and were sequenced using 454/Roche GS-FLX sequencer. Over 202,000 sequence reads (ENA accession no. ERS382901) analysed from the inoculum sample showed no additional nucleotide insertions at HA1/HA2 cleavage site and all cleavage site sequences were identical to LPAI Italy/1279 virus. However, analysis of 163683 sequence reads (ENA accession no. ERS381851, ERS381852, ERS381853, ERS382322 and ERS382902) made from tissue samples from dead turkeys showed a low level (overall 0.12%, range 0% to 0.5%) of sequence heterogeneity, possessing a 12 nucleotide insertion that encodes a polybasic amino acids (SRVR) identical to those that were present in H7N1 HPAI viruses isolated from poultry during the Italy 1999/2000 outbreak. The tissue samples that contained sequence reads with the additional 12 nucleotides were tracheal tissue from turkey #22 (0.025% of the reads) and liver (0.009% of the reads) and tracheal tissue (0.543%) from turkey #83 (Table 3), however the tracheal sample from turkey #95 showed no evidence (out of 38421 reads) for this insertion. Two additional minor variant sequences were also observed. They contained either one additional nucleotide (G) in 0.19% of sequences reads or two additional (GG) nucleotides in 0.01% sequences reads at the region of the HA gene encoding the cleavage site of the LPAI virus. These insertions of a single G or a double G were seen in all samples from turkeys #83 and #95 but were not observed in the tracheal samples from turkey #22. Addition of the single and double nucleotides resulted in a frame shift of the open reading frame of HA and thus viable virus could not have been generated carrying the corresponding HA genes (Table 3). No other variation was seen at the HA cleavage site.

**Table 3:** Ultra-deep amplicon sequence analysis of inoculum and tissue samples collected from turkeys that died or were culled following Italy/1279 H7N1 virus infection.

Bird ID	Died or culled atdays pi	Sample	Total numberof reads	Number of readsidentical to HPAIvirus* (%)	Number of reads withan additionalnucleotide (G) insertionat the cleavage site (%)	Number of reads withtwo additionalnucleotide (G) insertionat the cleavage site (%)	ENA sampleaccession number
		Inoculum	202665	0 (0)	0 (0)	0 (0)	ERS382901
22	9	Trachea	19975	5 (0.025)	0 (0)	0 (0)	ERS381851
83	4	Liver	32501	3 (0.009)	61 (0.187)	10 (0.031)	ERS381852
		Brain	36517	0 (0)	77 (0.210)	5 (0.014)	ERS381853
		Trachea	36269	197 (0.543)	100 (0.275)	2 (0.005)	ERS382322
95	9	Trachea	38421	0 (0)	76 (0.197)	5 (0.013)	ERS382902

* Amplicon reads with 12 additional nucleotide (TCGCGTGTGAGG) at the cleavage site encoding the signature SRVR amino acid sequence of the 1999/2000 HPAI H7N1 viruses in Italy.

## Discussion

The aim of the study described here was to investigate whether there was selective pressure that might result in the generation of HPAI viruses during replication of a LPAI virus within an infected host. The model based on infection of turkeys with a H7N1 LPAI virus from Italy collected in 1999 was chosen for several reasons. First, this outbreak has been studied in detail [15], [35] and during the outbreak virus variants had emerged, ultimately resulting in the circulation of a HPAI virus [16]. Second, turkeys are highly susceptible to avian influenza viruses and display varying disease signs [36]–[38]. Third, we observed that infection of turkeys with a LPAI H7N1 virus of chicken origin from Italy collected in 1999 resulted in serious disease signs and death following intranasal inoculation [28]. We speculated that within the infected turkeys the evolution of a highly pathogenic form of virus could have occurred. In light of reports that HPAI viruses can be detected under certain tissue culture passage conditions in the laboratory [21], [22], [24] and notably by propagation in older-aged embryonated fowls’ eggs [23], [25], [26], [39], we reasoned that virus replication in turkeys may impose similar selection pressures.

To examine the possibility that evolution of HPAI virus might have occurred in the infected host we investigated various explanations. The first possibility was that the virus stock used for the infection of turkeys could have contained a low titre of HPAI virus as a contaminating sub-species. We sequenced over 180 cDNA clones of the inoculum and found no evidence for the presence of a low level of HPAI virus and this was backed up by quantitative dRT-PCR and NGS in which no HPAI virus signature was detected in over 200,000 NGS reads.

Next we asked whether we could detect the evolution of HPAI virus from LPAI virus within the infected host. We examined over 3592 discrete cDNA samples from buccal and cloacal swabs by deep-amplicon sequence analysis and failed to detect any evidence of HPAI virus. To examine the possible generation of HPAI virus within the infected host, we examined tissues from dead or culled birds for evidence of a sequence signature of an HPAI virus. By deep-amplicon analysis of a total of 116 discrete cDNA clones we also found no evidence for HPAI virus in the tissues of infected birds. Similarly, no HPAI virus-specific sequence was detected with our dRT-PCR assay, capable of detecting very low levels of HPAI virus RNA with the HA polybasic cleavage site sequence signature that was observed in HPAI viruses isolated during the 1999–2000 poultry outbreak in Italy. We estimated that HPAI virus was not detectable by deep-amplicon analysis at around 0.12%; this represented less than 100 copies of HPAI HA gene equivalents in 10^6^ EID_50_ in the inoculum, and less than 100 DNA equivalent copies was detected in any tissue sample from the culled or dead birds by dRT-PCR analysis. The differential real-time RT-PCR would not detect viruses other than those for which we specifically designed the primers and TaqMan probes so analysis by NGS, using the Roche 454 sequencing platform, was used which allowed us to carry out deep-amplicon sequencing to a much greater depth and without any bias in the detection of sequences at the cleavage site. The NGS amplicons made from tissue samples contained a small number of reads (0 to 0.5%) with sequences containing a HPAI virus signature (Table 3). These were seen in the analysis of the cDNAs prepared from the tracheal sample of two of the three birds (0.025% and 0.5% of the sequences carried the HPAI virus signature) and in the liver sample from one of the birds (0.009% of the sequences carried the HPAI virus signature). No such signature was seen in cDNAs prepared from the tracheal sample from one bird or in cDNAs prepared from the brain sample from a bird in which the signature was seen in cDNAs prepared from the other two tissues examined. It is striking that these sequences at the HA cleavage site were identical to the residues observed in viruses isolated from the poultry outbreak in Italy during 1999–2000. Therefore either these HPAI virus sequence signatures have evolved from LPAI virus during replication in turkeys in an identical manner to that observed during the epizootic, or they represent very low contaminants of the LPAI virus sample. Importantly, no alternative HPAI virus sequence signatures were observed although a small number of reads with one- or two-base insertions at the cleavage site were detected: an insertion of a single nucleotide in 0.19% of the reads was observed and the insertion of two nucleotides in 0.01% of the reads. We do not know if these occur as a sequencing artefact or represent intermediates in the evolution of an HPAI virus, but it is noteworthy that no insertions were observed at the cleavage site of the inoculum, suggesting that these insertions in the sequences of the HA gene do not represent systematic sequencing errors. We can reach no firm conclusion as to the origin of the 205 reads of virus with a HPAI virus signature: these sequences were not detected in the inoculum, and due to their unique sequence and their identity with HPAI virus evolving at the time of the collection of the low pathogenicity virus Italy/1279, we presume that they are a contaminant of the inoculum that was preferentially amplified during replication in the turkey. To reiterate, the failure to observe alternative insertions of nucleotides encoding additional amino acids at the HA1/HA2 cleavage site indicate that evolution of HPAI virus signature sequences at this site is likely to be a very infrequent occurrence.

It is well established that viruses without a series of basic amino acids at the HA1-HA2 cleavage site can result in an elevated IVPI [7], [40], [41] and viruses with a low IVPI can cause death following intracerebral inoculation [42]. Swayne and Alexander detected high titres of virus in the kidneys in most chickens following infection by the intravenous route and in these birds gross and histopathological lesions in the kidney were observed along with the detection of virus in kidneys and pancreatic acinar cells [41]. A histopathological study of the turkeys could not be undertaken during this work but studies on the clinical, gross and microscopic findings in turkeys carried out during the outbreak of infection with H7N1 in Italy during 1999 reported widespread lesions in the internal organs, including the lungs and pancreas, together with egg-yolk peritonitis in breeder turkey flocks [43]; therefore, it is likely that viruses similar to Italy/1279 were particularly virulent in turkeys prior to the evolution of the viruses with a HPAI virus pathotype [44].

It is pertinent that in the vast majority of primary outbreaks of HPAI virus, the low pathogenicity precursors have not been directly detected, although some may be adduced as evolutionary precursors retrospectively [45]. However, in the Italian 1999–2000 epidemic the LPAI form of virus was detected in turkeys nine months before the documented emergence of HPAI virus [16]. It is notable that the recognition of the emergence of HPAI virus from a LPAI virus in domesticated poultry resulted in a change in legislation in the European Union that made all infections caused by H7 and H5 avian influenza viruses notifiable under the EU Council Directive 2005/94/EC [46] and were consistent with revisions in the OIE definition [29].

## Supporting Information

Table S1 **Virus RNA titres in buccal and cloacal swabs collected over 8 days from turkeys inoculated with 10^5.2^ EID_50_ Italy/1279 H7N1 virus.**
(DOCX)

Table S2 **Virus RNA titres in buccal and cloacal swabs collected from turkeys that died following Italy/1279 H7N1 virus infection whose tissues were selected for deep amplicon sequence analysis.**
(DOCX)
