# Supplementary material for: Virus Pathotype and Deep Sequencing of the HA Gene of a Low Pathogenicity H7N1 Avian Influenza Virus Causing Mortality in Turkeys
Source: PLoS One. 2014 Jan 28;9(1):e87076. doi: 10.1371/journal.pone.0087076 (PMC3904975; doi:10.1371/journal.pone.0087076)
Supplement: Table S1 — Virus RNA titres in buccal and cloacal swabs collected over 8 days from turkeys inoculated with 105.2 EID50 Italy/1279 H7N1 virus. (DOCX) [file pone.0087076.s001.docx]

**Table S1. Virus RNA titres in buccal and cloacal swabs collected over 8 days from turkeys inoculated with 10 ^5.2^ EID_50_ Italy/1279 H7N1 virus.**

|  | | Days post infection | | | | | | | | |
| --- | --- | --- | --- | --- | --- | --- | --- | --- | --- | --- |
| Turkey ID | Swab | 1 | 2 | 3 | 4 | 5 | 6 | 7 | 8 | 9 |
| 21 | Buccal | 2.48 | 3.9 | 3.62 | 2.59 | Died |  |  |  |  |
|  | Cloacal | - | - | - | - |  |  |  |  |  |
| **22** | Buccal | 1.65 | 3.27 | 2.86 | 1.32 | 2.68 | 2.43 | 3.47 | 2.46 | Killed |
|  | Cloacal | - | - | - | - | - | - | 1.56 | 1.0 |  |
| 23 | Buccal | 1.89 | 1.33 | 2.76 | 3.48 | 2.47 | 2.27 | 3.13 | 3.25 | Killed |
|  | Cloacal | - | - |  | - | - |  | 2.73 | 0.3 |  |
| 24 | Buccal | 2.05 | 4.13 | 3.34 | 2.65 | 2.86 | 2.26 | Died |  |  |
|  | Cloacal | - | - | 0.3 | 1.14 |  | - |  |  |  |
| 25 | Buccal | - | - | - | 1.90 | 2.51 | 2.62 | Died |  |  |
|  | Cloacal | - | - | - | 3.01 | 1.28 | 1.81 |  |  |  |
| 26 | Buccal | 3.27 | - | - | - | Died |  |  |  |  |
|  | Cloacal | - | - |  | - |  |  |  |  |  |
| 27 | Buccal | 1.05 | 1.94 | 4.15 | 3.31 | 2.36 | Died |  |  |  |
|  | Cloacal | - | - | 5.49 | 1.17 | 1.67 | - |  |  |  |
| 28 | Buccal | 2.46 | 2.94 | 3.54 | 2.75 | 2.19 | 2.05 | 1.80 | 1.29 | Killed |
|  | Cloacal | - | - | 1.01 | - | - | 1.29 | 2.66 | 2.02 |  |
| 29 | Buccal | 4.41 | 3.69 | 3.49 | 3.17 | Died |  |  |  |  |
|  | Cloacal | - | - | 1.08 | 3.77 |  |  |  |  |  |
| 30 | Buccal | 2.02 | 4.0 | 3.04 | 2.62 | 2.96 | 2.54 | 2.68 | Died |  |
|  | Cloacal | - | - | - | - | - | 2.02 | 1.89 |  |  |

Viral RNA levels in swabs were detected by real-time RT-PCR. Measured Ct values were extrapolated as relative equivalent units (REU) of virus infectivity titres in log_10_ EID_50_ per ml based on a standard curve constructed from RNA extracted from known EID_50_ titres of Italy/1279. (-) indicates RNA titres <10^1^ EID_50_ per ml. Tissue samples from turkey #22 (highlighted in bold) were used for deep amplicon sequence analysis.
