# Supplementary material for: Virus Pathotype and Deep Sequencing of the HA Gene of a Low Pathogenicity H7N1 Avian Influenza Virus Causing Mortality in Turkeys
Source: PLoS One. 2014 Jan 28;9(1):e87076. doi: 10.1371/journal.pone.0087076 (PMC3904975; doi:10.1371/journal.pone.0087076)
Supplement: Table S2 — Virus RNA titres in buccal and cloacal swabs collected from turkeys that died following Italy/1279 H7N1 virus infection whose tissues were selected for deep amplicon sequence analysis. (DOCX) [file pone.0087076.s002.docx]

**Table S2. Viral RNA titres in buccal and cloacal swabs collected from turkeys that died or were culled following Italy/1279 virus infection whose tissues were selected for deep amplicon sequence analysis**.

| Turkey ID | Infection route (dose EID_50_) | Swab | Days post infection | | | | | | | | |
| --- | --- | --- | --- | --- | --- | --- | --- | --- | --- | --- | --- |
|  |  |  | 1 | 2 | 3 | 4 | 5 | 6 | 7 | 8 | 9 |
| 83* | Inoculated (10^3.8^) | Buccal | 4.1 | 4.2 | 4.1 | Died |  |  |  |  |  |
|  |  | Cloacal | - | - | 4.3 |  |  |  |  |  |  |
| 95* | Contact | Buccal | - | 2.5 | 4.3 | 5.0 | 4.0 | 3.1 | 3.2 | 2.3 | Died |
|  |  | Cloacal | - | - | - | - | - | - | - | - |  |

The contact turkey #95 was housed with inoculated turkey #83. Viral RNA levels in swabs were detected by real-time RT-PCR. Measured Ct values were extrapolated as relative equivalent units (REU) of virus infectivity titres in log_10_ EID_50_ per ml based on a standard curve constructed from RNA extracted from known EID_50_ titres of Italy/1279 virus. (-) indicates RNA titres <10^1^ EID_50_ per ml. * Data published in reference 28.
